# Diet and Health Benefits Associated with In-Home Eating and Sharing Meals at Home: A Systematic Review

**DOI:** 10.3390/ijerph18041577

**Published:** 2021-02-07

**Authors:** Karen Glanz, Jessica J. Metcalfe, Sara C. Folta, Alison Brown, Barbara Fiese

**Affiliations:** 1 Perelman School of Medicine and School of Nursing, University of Pennsylvania, Philadelphia, PA 19104, USA; 2 Department of Food Science and Human Nutrition, University of Illinois, Urbana, IL 61820, USA; jarick2@illinois.edu; 3 Friedman School of Nutrition Science and Policy, Tufts University, Boston, MA 02111, USA; sara.folta@tufts.edu; 4 National Heart, Lung and Blood Institute, NIH, Bethesda, MD 20892, USA; alison.brown@nih.gov; 5 Department of Human Development and Family Studies, University of Illinois, Urbana, IL 61820, USA; bhfiese@illinois.edu

**Keywords:** in-home eating, shared meals, healthy diets, family mealtimes, home meals

## Abstract

In-home and shared meals have been hypothesized to have positive effects. This narrative review examines research on the influence of in-home eating on diet quality, health outcomes, and family relationships. A combination search approach included a search of PubMed, backward searches of previous published reviews, and studies the authors were familiar with. A search identified 118 publications; 54 original studies and 11 review studies were included in this review. Each study was reviewed and summarized. The diverse designs precluded quantitative data synthesis. Relatively strong evidence from cross-sectional research supports the association of shared family meals with favorable dietary patterns in children and adolescents, including consumption of fruits, vegetables, and healthful nutrients. Correlational evidence links shared meals with health and psychosocial outcomes in youth, including less obesity, decreased risk for eating disorders, and academic achievement. Most evidence is cross-sectional, thus, limiting attribution of causality. There is insufficient evidence to conclude that interventions improve the frequency of shared meals, improve diet, or prevent child obesity. Despite the “common wisdom”, the evidence that in-home, shared meals, per se, have positive effects on diet quality, health outcomes, psychosocial outcomes, and family relationships is limited due to weak research designs and single-item measurement of the independent variable. More research, with stronger designs, is warranted.

## 1. Introduction

Evidence suggests that there have been shifts in dietary practices over the past few decades, with less time devoted to food shopping, cooking, and in-home eating despite the potential benefits of in-home food preparation and eating [1]. Paralleling this decrease in time spent preparing food in the home and changes in diet is the worldwide increase in diet-related health concerns such as obesity and diabetes [2,3].

In light of these trends, health experts are seeking effective strategies to combat the obesity epidemic, including the possible role of in-home and family-shared meals. Some research suggests a potential protective effect of home meals on child health, psychosocial outcomes, and family relationships [4]. For example, dining together in the home has been proposed to foster self-esteem, promote academic achievement, and protect against substance abuse in adolescents [5,6,7]. To date, however, there is no available review of current evidence for benefits of in-home meals on diet quality and meal patterns, health outcomes, psychosocial factors, and family relationships.

The purpose of this narrative literature review is to examine the evidence regarding the effects of in-home eating and shared meals. Specifically, the review examines the scientific literature on: (1) factors associated with in-home eating (e.g., lifestyle habits, family demographics); (2) the impact of in-home eating on the nutritional quality of meals; (3) the relationship between in-home eating and child and adolescent health outcomes; and (4) the influence of in-home eating on family relationships.

## 2. Materials and Methods 

**Figure 1:**
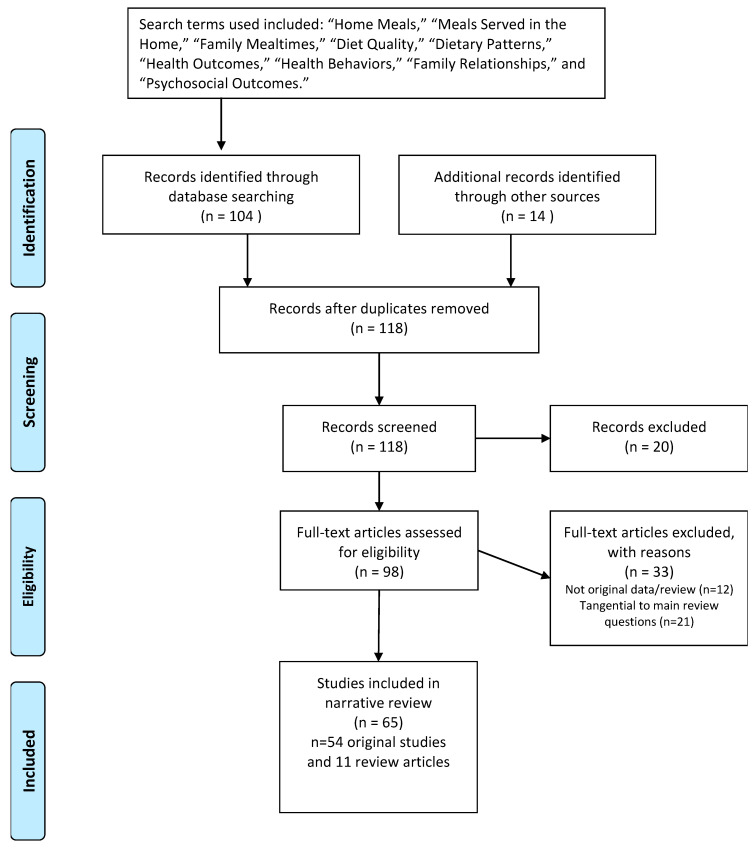
Flow Diagram.

Primary data-based articles and review articles were included in this literature review, which was initially conducted between January and April 2016. Search terms used included: “Home Meals”, “Meals Served in the Home”, “Family Mealtimes”, “Diet Quality”, “Dietary Patterns”, “Health Outcomes”, “Health Behaviors”, “Family Relationships”, and “Psychosocial Outcomes”. A combination search approach was used that included a search of the PubMed database, backward searches of previous published reviews on the topic, and studies the authors were familiar with based on their combined 25 years of research in the area. In the initial literature search, 112 publications were identified and of these, 48 original studies and 11 additional review publications were considered suitable to include in this review. Publications were excluded if they did not report on original data or a systematic review on this topic, or if they were found to be tangential to the main review question. An updated search in May 2018 yielded six additional relevant primary research articles. Analysis of the literature was completed in 2018–2020 (See Figure 1).

Studies were organized into categories based on the review questions. Most of the studies were conducted in the U.S., but we have noted non-U.S. study locations in the Results tables where applicable. A preliminary scan revealed the literature is comprised of mostly cross-sectional studies, a variety of methods and measures, and few randomized controlled trials. This limited the ability to apply formal statistical comparisons to the body of literature, so a narrative review approach was chosen, whereby the review team members would each examine studies within outcome categories, summarize their observations, and discuss the findings with the team until consensus was reached. Narrative reviews are preferred when the body of research cannot be summarized quantitatively due to the heterogeneity of research methods and statistical analyses [8,9].

### 2.1. Terminology

#### 2.1.1. Defining and Measuring In-Home Meals and Shared Meals at Home 

Most of the research in this area focuses on in-home meals or shared meals at home. The concept of “in-home meals”, or in-home eating—without mention of shared meals or family meals—has often been studied in the context of comparing meals eaten at home versus meals eaten outside the home [10,11]. *Shared meals at home*, or *frequency of family meals* is the focus of a greater number of studies [4,12]. Most use a similar approach to measuring this construct, namely a single item with this wording or similar wording: “During the past week, how many times did all, or most, of the people living in your household eat a meal together?” [5,7,13,14,15,16,17,18,19,20,21,22,23,24] Some of these studies ask whether “the family typically eats together” for various meals [25], or “how often do you eat dinner with a parent or guardian?” [26]. The question is usually asked of parents and/or children and adolescents. Most of the measures focus on dinner or the evening meal. Response options are usually specified by the number of days per week (0 to 7) or using ordinal labels, such as ‘never/rarely’ to ‘always’ [26,27,28].

#### 2.1.2. Other Behaviors and Circumstances Closely Related to In-Home Meals and Shared Meals at Home 

The two most-often measured behaviors and circumstances, related to in-home meals and shared meals at home (but not measuring meals per se), are *home food preparation/cooking* and *social interaction during meals* at home. In their protocol for a systematic review of home cooked meals, Mills et al. [29] defined *home cooking* as “practices and skills for preparing hot or cold foods at home, including mixing, combining, and often heating ingredients.” The Project EAT cohort study included a question about “frequent food preparation in the home” [12] and other studies ask how many days per week the child “eats a dinner that was made at home” [21].

Assessments of *social interaction during meals* at home include items such as the presence of an adult at the meal, eating in the kitchen or dining room, and watching TV while eating [30,31]. Several studies have used videotaped interactions to assess social interactions, such as feeding styles, interaction, and communication during family meals [32,33,34,35].

## 3. Results

### 3.1. Diet Quality and Meal Patterns

We examined the available studies to summarize whether in-home eating and meal sharing are associated with energy intake, fruit and vegetable intake, nutrient intake, dietary patterns, and overall diet quality in children and adolescents. The majority of the studies to date have been cross-sectional (16), and a smaller number are longitudinal (4). In addition, four systematic reviews have been published. Overall findings suggest a positive association between the frequency of family meals and favorable dietary outcomes (Table 1).

Project EAT, a longitudinal study conducted in the U.S. Midwest, has contributed substantially to the literature regarding the influences of family meals on the dietary outcomes of adolescents [13,15,36,37]. In our review, five empirical research reports drew from the Project EAT dataset and all four systematic reviews included Project EAT findings. It is noteworthy that most studies used intakes of specific food groups, typically fruit and vegetable consumption, as a proxy for diet quality; and only one used a validated measure of overall diet quality (the Diet Quality Index–International) [38]. Additionally, while most of the research was conducted in the U.S., a portion of the literature is based on studies in Europe and other nations outside of the U.S. [10,38,39,40,41,42]. The conclusions mostly apply to the U.S. cultural context and food environment.

Family meal frequency is positively associated with fruit and vegetable and dairy intakes and breakfast eating; and negatively associated with fried foods, unhealthy snacks and cakes, and sugar-sweetened beverage intake [12,13,14,37,39,44,45,47,50,51,52,53,54]. The impact of family meals on specific foods may differ by age. In a cross-sectional study of 1992 children (age 0 to 19 years), five or more family meals per week was associated with lower sugar-sweetened beverage intake among both younger and older children, greater vegetable intake among older children and adolescents, and greater fruit intake among adolescents [18]. However, one U.K.-based study found that the frequency of family mealtimes was unrelated to vegetable consumption or liking among children ages 2 to 5 years old [41].

Family meal frequency has been shown to be positively associated with increased intakes of calcium, fiber, magnesium, potassium, iron, zinc, folate, thiamin, riboflavin, B12, B6, and vitamins A, C, and E [12,13,19,25,37,40,46,47,52]. The evidence regarding caloric intake is inconsistent. Neumark-Sztainer, Hannan et al. [13] found a positive association between frequency of home meals and energy intake in a study of 4746 adolescents. However, a longitudinal nationally representative sample of 29,217 children in the U.S. found that between 1977 and 2006, there was an overall increase in energy intakes and a corresponding decrease in frequency of eating at home [48].

The influence of family meals on dietary outcomes may also differ by sex. Burgess-Champoux, Larson et al. [15] found that regular family meals had a positive association with increased sodium intake for females, but not males. Finally, there may be different outcomes by race/ethnicity. A recent study found a positive association between an increase in family meal frequency over time and fruit and vegetable consumption in the eighth grade among white and black adolescents, but not among Hispanic or Asian adolescents [49].

### 3.2. Health Outcomes

The majority of studies that examine associations between sharing meals together and health focus on outcomes that are most closely tied to nutrition (Table 2). Using large nationally representative datasets, direct relationships between frequency of shared meals and reduced risk for childhood overweight and obesity are frequently reported [20,55,56,57]. These findings are confirmed in meta-analytic reviews of smaller studies [45]. However, when more sophisticated modeling techniques are used to include potential confounders or mediators, such as family cohesion and closeness, the direct link between mealtime frequency and child obesity often is minimized or becomes nonsignificant [50]. Further, when racial and ethnic differences are carefully examined, nonmonotonic differences are revealed [58] and some studies found no associations between meal frequency and risk of overweight for Hispanic families [58,59].

Eating disorders and restricted eating habits, such as extreme dieting, have also been examined in relation to sharing meals together. In general, a significant relationship between eating disorders and low levels of sharing meals together has been reported [60,61,62]; but these effects are often minimized when family factors such as stability are included in analyses [61].

**Table 2:** Summary of Health Outcomes.

Author(s) and Year	Reference Number	Sample	Main Outcomes	Study Design
Health Outcomes				
Anderson and Whitaker (2010)	[20]	8850 children aged 4	Children who ate dinner with their families more than five nights per week were less likely to be obese.	Cross-Sectional
Berge et al. (2014)	[33]	120 children aged 6–12 from low-income and minority communities	Positive family and parent-level interpersonal dynamics during family meals were associated with reduced risk of children being overweight. Positive family and parent-level food-related dynamics during family meals were associated with reduced risk of childhood obesity.	Cross-Sectional
Fiese et al. (2012)	[32]	200 families with children aged 5–12	Families with overweight or obese children spent less time on a family meal and spent less time in positive communication than families with children of a healthy weight.	Observational
Franko et al. (2008)	[28]	2379 females tracked annually from age 9–19	More frequent family meals from ages 9–11 predicted lower likelihood of bulimic symptoms, drive for thinness, and smoking behaviors during adolescence. Family cohesion mediated the relationships between family meals and risk of smoking behaviors.	Longitudinal
Gable et al. (2007)	[55]	8549 children were followed from kindergarten through third grade	Children who ate fewer family meals during kindergarten and first grader were more likely to be overweight in third grade.	Longitudinal
Hammons and Fiese (2011)	[45]	182,836 children and adolescents across 17 studies	Children and adolescents who ate meals with family 3 or more times per week had healthier weight status than those who ate fewer than 3 meals with family per week.	Meta-Analysis
Jones et al. (2014)	[63]	337 preschool-age children	Children who participated in frequent family meals were more likely to get 10 or more hours of sleep per night.	Cross-Sectional
Kitzman-Ulrich et al. (2010)	[64]	Youth (elementary age–adolescents) who participated in weight loss, physical activity, or dietary interventions	Family functioning and parenting styles should be investigated as potential mediators of intervention outcomes in weight loss and physical activity interventions.	Systematic Review
Loth et al. (2015)	[62]	2382 middle and high school students, Project EAT	Greater frequency of family meals was associated with decreased odds of engaging in unhealthy weight control behaviors in boys, and dieting, unhealthy and extreme weight control behaviors in girls.	Cross-Sectional
McCurdy et al. (2014)	[31]	164 low-income, preschool-aged children and their mothers	Maternal presence when the child ate was associated with lower BMI z-scores for children.	Cross-Sectional
Munoz et al. (2007)	[61]	134 female undergraduate students	Overall family stability was a more comprehensive predictor or bulimia symptom than family meal frequency alone.	Cross-Sectional Retrospective
Neumark-Sztainer et al. (2008)	[60]	2516 adolescents, Project EAT	Adolescent girls who ate five or more meals per week with their families in middle school were less likely to engage in extreme weight control behaviors five years later.	Longitudinal
Piazza-Waggoner, et al. (2011)	[65]	56 families with obese and non-obese children	Parents and caregivers of obese children reported greater mealtime challenges and a less positive mealtime environment than caregivers of non-obese children. There were no differences in observed mealtime interactions in families with obese and non-obese children.	Observational
Rollins et al. (2010)	[56]	16,770 children aged 6–11 (national sample)	Family meals were protective against obesity in non-Hispanic White children and non-Hispanic Black boys, were a marginal risk factor for obesity for Hispanic boys living in low-education households.	Cross-Sectional
Sen (2006)	[58]	5041 youth aged 12–15 (national sample)	Family meal frequency at age 12 was associated with healthier weight status at age 12 and age 15 for White participants. No associations between family meal frequency and weight status for Black and Hispanic participants.	Cross-Sectional
Skeer and Ballard (2013)	[57]	Families with adolescent children	Frequent family meals were associated with decreased risk for overweight and obesity in females.	Literature Review
Taveras et al. (2005)	[59]	14,431 children aged 9–14 (national sample)	Children who ate dinner with their families most days or everyday were less likely to be overweight than children who ate dinner with their families never or some days.	Longitudinal
Utter et al. (2008)	[50]	3245 adolescents (national sample)	Frequency of family meals was not significantly related to BMI when demographics were included in the model.	Cross-Sectional
Wansink and van Kleef (2014)	[30]	190 parents and 148 children in third-sixth grade	Families who regularly ate dinner in the dining room or kitchen had lower BMIs (for both adults and children). Helping cook dinner was associated with higher BMI for females and remaining at the table until everyone is finished eating was associated with lower BMI for males.	Cross-Sectional

Beyond diet quality, it is possible that social interaction during meals may mediate or contribute to some of the health outcomes reported [64]. Direct observations of social interactions during in-home meals have found that positive communication, showing genuine concern about family daily activities, and length of meal is associated with reduced risk for overweight and obesity [32,33]. However, treatment-seeking families (e.g., those seeking to lose weight) do not show this pattern when compared to families of non-obese children [65]. Presence of an adult and eating in the kitchen or dining room has also been found to be associated with less risk for being overweight or obese [30,31].

A consistent criticism in this area of research is whether shared mealtime is a proxy for other factors such as good parenting and positive family climate. Some research controls for these factors and finds decreased effect sizes in predicting health outcomes [28]. Further, mealtime routines are often highly related to other family routines, such as sleep routines [20,63], which are known to affect health outcomes.

### 3.3. Psychosocial Outcomes

A number of studies have investigated the association between the frequency of shared family meals and specific psychosocial characteristics and risk behaviors (Table 3). Positive associations were found between the frequency of family meals and self-esteem, academic achievement, commitment to learning, positive perceptions of support and boundaries, positive values, social competencies, and overall psychological well-being [5,6]. Negative associations were found between the frequency of family meals and substance use (alcohol, tobacco, and illicit drugs), depressive symptoms, suicidal ideation, eating disorders, unhealthy and extreme weight-loss practices, and violence/delinquency [7,14,57].

The benefits of family meals appear to vary somewhat by gender, especially for adolescents. Regular family meals during middle school were found to be associated with decreased odds of tobacco, alcohol, and drug use five years later, but only for females [7]. Sen [66] found that the frequency of family meals was related to decreased substance use and running away for females, and decreased incidence of drinking, physical violence, property destruction, stealing, and running away from home for males. Benefits of family meals in relation to healthy eating behaviors appear to be stronger for female adolescents; frequent family meals were associated with lower prevalence of “extreme weight control behaviors” in females, [60,62] decreased bulimic symptoms, and lower drive for thinness [28].

Most of the studies that included psychosocial variables were cross-sectional and focused on adolescents. Almost all of the data suggesting a relationship between family meals and psychosocial outcomes relates to the quantity or frequency of family meals, without assessing the quality of these eating experiences. One exception is a study by Neumark-Sztainer, Wall, and others [68], which found that more structured family meal environments and a positive atmosphere at family meals were associated with decreased odds of disordered eating. One study that used experience sampling methods found that meals eaten at home were followed by more positive emotions and less worry than meals eaten away from home [67].

Although most studies controlled for family demographic characteristics, only two [7,28] included measures of family functioning, such as family connectedness and cohesion. A review by Goldfarb and colleagues [6] concluded that associations between the frequency of family meals and deceased risk behaviors are less likely to be significant when factors like family connectedness are considered, indicating that family functioning may be more responsible for these associations than family meals themselves.

### 3.4. Family Relationships

Sharing meals together as a family has been proposed to promote relational closeness and to be a family routine that may promote health and wellbeing [32,69]. The mechanisms are believed to occur through repeated social interactions whereby emotions are well-regulated, positive parenting is demonstrated, and there is relatively little open conflict. Cross-sectional studies linking overall family functioning and the frequency of family mealtimes have generally found positive connections [24,28,54,70] (Table 4).

A recent longitudinal report [26] suggests that dining together may actually encourage better communication skills in adolescents. Over a 3 ½ year period, the investigators found that frequent family dinners during early adolescence predicted parent–child communication frequency over time. Those families who started out with higher mealtime frequencies had adolescents who reported less decline in parent–child communication during the early adolescent years. This is one of the few longitudinal studies that examined the predictive effects of mealtime frequency on family dynamics over time.

### 3.5. Correlates of In-Home Eating and Shared Meals

Sociodemographic characteristics, including gender, household income, race/ethnicity, and household size have been hypothesized to be correlates of in-home dining and potential influences on the effects of in-home eating on a variety of outcomes. Some studies [14,71] did not find any significant associations between the frequency of family meals and sociodemographic characteristics (e.g., gender, race/ethnicity, socioeconomic status, and family structure), but other research has found data that link these factors to in-home eating.

For example, both parent and child gender seem to be related to in-home eating and food preparation habits. Nearly four times as many women as men reported cooking every day (68 vs. 18 percent) [73]. One study found that the participation in family meals decreases as adolescents grow older [66], and another study found this to be true only for females [26].

Income has been found to inversely relate to *home food consumption*, as individuals with lower incomes consume more of their diets through home food sources [74]. Regarding *shared family meals*, there is evidence that families with high parental income and education are more likely to eat meals together as a family [63], while families who experienced financial strain may be less likely to eat together [21].

Two-parent biological families and families with two or more children have been found to be more likely to have frequent family meals [72,75]. These data support the idea that single parenting may be more important than income as an obstacle to shared family meals [76]. The evidence is mixed regarding the associations between race/ethnicity and family meal frequency [71]. One study found that Hispanic and White families eat dinner together *more* frequently than Black families [19], while another study found that minority (Hispanic and Black) children were *less* likely to eat lunch or dinner with their families than White children [43]. However, much of the research to date has focused on nonrepresentative convenience samples, so the generalizability of the findings are difficult to ascertain.

### 3.6. Interventions to Increase Shared Meals at Home

There are four published studies to date that report on evaluations of intervention strategies to increase shared meals at home. We used the GRADE tool [77] for assessing risk of bias to evaluate the certainty of estimated effects found in these studies. The first of these studies, by Johnson and others [78], evaluated a family meals module in Women, Infants, and Children (WIC) program clinics across Washington State, and compared it to a family activity module. The intervention module (or “package”) included background information, training for WIC staff, group sessions, print materials, and collateral materials (bookmarks, coloring sheets, etc.). The program was implemented in 59 clinics, and 7 to 82% of clients at each clinic received the program. The outcome evaluation involved randomization of clinics and compared cross-sectional surveys at program (intervention) and control clinics before and after the program was offered; however, the study could not track individual participants. There was a significant increase in meals eaten together in the program groups, but the impact was very small (less than ½ meal increase per week) because the baseline rate of shared meals at home was already quite high (5 to 6 days per week).

The second intervention study reported was the HOME Plus Study conducted by Fulkerson et al. [79]. This trial aimed to promote healthful family meals to prevent obesity and included 160 8 to 12 year-old children. The intervention was a 10-session program of nutrition education, meal and snack planning, food preparation, skill development, and screen-time reduction; plus five tailored, goal-related motivational phone calls. The main outcome was child BMI z-score. Results found no significant treatment group differences in child weight/obesity, although there was a modest decrease in excess weight gain due to the intervention. Most of the participants were not obese at baseline, and the study had a relatively small sample. The study found no significant effect of the program on frequency of family meals. Most participants (60 to 70%) ate five or more family meals together per week at baseline, so there was little room for change.

A pilot study [80] and, therefore, not included in this panel’s initial scan of the literature, tested the effects of a parent–child cooking program on decreasing meals eaten away from home. The evaluation, using a pre-post test design with no control group, found that the cooking intervention achieved a significant effect of decreasing eating-out. This finding has promise; however, the study included only six families and did not have a control group for comparison. Another pilot study, of the iCook 4-H program [81], found no significant increases in family meal frequency because the baseline rate of shared meals was already high, similar to the Johnson et al. [78] and Fulkerson et al. [79] studies described above.

Our findings using the GRADE tool for assessing risk of bias to these four studies reveal the limitations of this intervention literature. One study (Fulkerson et al., [76]) had moderate certainty (but found no significant effects on shared meal frequency or weight), two studies had low certainty [75,78], and one had very low certainty [77].

## 4. Discussion

Most of the published research on in-home meals focuses on the relationships between frequency of sharing meals together in the home and outcomes such as dietary patterns and psychosocial outcomes. There is relatively strong evidence from cross-sectional research suggesting that greater frequency of sharing meals together as a family is associated with favorable dietary patterns in children and adolescents, including consumption of fruits and vegetables and many nutrients. There is also a preponderance of correlational evidence linking shared meal frequency with health and psychosocial outcomes in children and adolescents including less obesity, decreased risk for eating disorders, and academic achievement. Most studies also found significant associations between socioeconomic status (SES), specifically household income and parental education and shared family meals. Paradoxically, those with lower incomes eat more of their food from home sources [69]. Moreover, two-parent biological families report more shared family meals. However, because most of the study designs are cross-sectional, they preclude causal inference. It is also not possible to disentangle the effects of race in this body of research, in part because race and SES may be intercorrelated. It also was not possible to apply formal statistical comparisons to this body of literature. A recurring question in the research is whether sharing meals together is a marker of positive parenting practices overall, or of family cohesion or structure.

There is insufficient evidence to conclude that intervention programs that include nutrition education, meal planning, and parent–child activities can improve the frequency of shared meals, improve diet, or prevent child obesity. Limitations in design and execution were substantial in three of the four available studies. A key limitation is that the few intervention studies in the literature enrolled participants with high family meal frequency at baseline.

A cross-cutting issue in the literature on family meal-sharing relates to how researchers measured the behavior of having *shared meals at home*. A key strength is that findings are relatively comparable across studies or publications. However, there are several limitations as well. First, the single item from self-report data is prone to social desirability bias and systematic error from memory limitations, and does not have the sensitivity to assess seasonal changes (e.g., school year versus summer) or changes over time. Second, there is a possible measurement effect of asking this question in the same cross-sectional surveys where respondents report on their food choices. Moreover, few studies ask about both meals eaten at home *and* meals eaten outside the home. Most studies asked this question of parents, while others asked children/adolescents; we found no available data about the concordance of parent vs child meal-sharing reports. This issue warrants future research. Finally, few studies have examined the (criterion) validity of this measure, with the exception of the study by Franko and colleagues [28], which found that girls’ responses to how often they ate with their families were validated by asking parents the same question.

## 5. Conclusions

Despite what is increasingly becoming “common wisdom,” the evidence that in-home, shared meals, per se, have direct positive effects on diet quality, health outcomes, psychosocial outcomes, and family relationships is greatly limited by research designs and measurement of the hypothesized independent variable. Measures should include how food is prepared and served in the home, not merely whether children and parents eat together, as well as the quality of meals. There is a need for more longitudinal studies that track changes in mealtime frequency and family dynamics over time to predict changes in diet quality, health outcomes, psychosocial outcomes, and family relationships; while incorporating appropriate controls for other factors that may predict key outcomes as well as meal-sharing. More adequately powered intervention studies and randomized trials are needed. Intervention studies that avoid “ceiling effects” by including mainly those who already share meals frequently are also needed. Further, there is a need to pay more attention to family diversity in terms of race/ethnicity, socioeconomic status, household composition, and parenting roles.

The emerging results suggest that shared meals and in-home eating may have protective effects against child and adolescent overweight/obesity. However, much more research, with stronger designs and more rigorous measurement of predictors, is warranted.

## Figures and Tables

**Table 1:** Summary of Diet Quality and Meal Patterns.

Author(s) and Year	Reference Number	Sample	Main Outcomes	Study Design
Diet Quality & Meal Patterns				
Ayala et al. (2007)	[25]	167 Mexican American children, 8–18 years old and their mothers	Number of family meals positively associated with fiber intake.	Cross-Sectional
Burgess-Champoux et al. (2009)	[15]	677 adolescents, Project EAT	Five or more family meals per week associated with increased sodium intake for females, but not males. Five or more family meals per week during the first wave of the study was associated with frequency of breakfast, lunch, and dinner meals for males, and only breakfast and dinner for females five year later.	Longitudinal
Burke et al. (2007)	[40]	594 Irish children, 5–12 years	Reported fiber and micronutrient intake were higher during eating occasions inside the home compared to outside of the home.	Cross-Sectional
Chu et al. (2014)	[38]	3398 Canadian children, 10–11 years	Higher frequency of involvement in home meal preparation was associated with higher diet quality index scores. Children who were involved in meal preparation daily ate 1 more serving/day of vegetables and fruit compared with children who never helped.	Cross-Sectional
Fink et al. (2014)	[18]	1992 children (age 0 to 19 years)	Five or more family meals per week associated with less sugar-sweetened beverage intake among younger and older children, greater vegetable intake among older children and adolescents, and greater fruit intake among adolescents.	Cross-Sectional
Fitzpatrick et al. (2007)	[19]	1336 parents of children aged 1–4 participating in WIC	Number of days per week the family ate dinner together was positively associated with serving fruit and serving vegetables.	Cross-Sectional
Flores et al. (2005)	[43]	2608 parents of children ages 4–35 months	Minority children less likely than whites to have consistent mealtimes, and more likely to never eat lunch or dinner with their families. The analyses also addressed home safety practices for young children, and found disparities with fewer practices in minority homes.	Cross-sectional
Fulkerson et al. (2009)	[14]	Racially diverse sample of 145 adolescents who attended alternative high school	Family dinner frequency was positively associated with breakfast consumption and fruit intake.	Cross-Sectional
Fulkerson et al. (2014)	[12]	Child, adolescent, or adult samples with findings related to family meals or commensal eating	Studies included in review found associations between family meal frequency and intake of fruits and vegetables, micronutrients, and breakfast, and decreased intake of soda, higher-fat foods, unhealthy snacks and cakes, fried foods, and fast food.	Systematic Review
Gillman et al. (2000)	[44]	16,202 youth aged 9–14	Eating family dinner was associated with consuming more fruits and vegetables, less fried food and soda, less saturated and trans-fat, lower glycemic load, more fiber and micronutrients from food, and no material differences in red meat or snack foods.	Cross-Sectional
Haapalahti et al. (2003)	[39]	404 Finnish children aged 10–11	Children who ate family dinner regularly consumed less fast food and sweets but more juice than children who did not have regular family dinners.	Cross-Sectional
Hammons and Fiese (2011)	[45]	182,836 children and adolescents across 17 studies	Children and adolescents who ate meals with family 3 or more times per week had healthier dietary patterns than those who ate fewer than 3 meals with family per week.	Meta-Analysis
Larson et al. (2006)	[36]	1710 young adults aged 18–23, Project EAT	Young adults who reported frequent food preparation reported less frequent fast-food use and were more likely to meet dietary objectives for fat, calcium, fruit, vegetable, and whole-grain consumption.	Cross-Sectional
Larson et al. (2007)	[37]	1710 young adults aged 18–23, Project EAT	Family meal frequency during adolescence predicted higher intakes of fruit, vegetables, dark-green and orange vegetables, and key nutrients and lower intakes of soft drinks during young adulthood.	Longitudinal
Martin-Biggers et al. (2014)	[46]	Families (with children)	More frequent family meals are associated with greater consumption of healthy foods in children, adolescents, and adults. Adolescents and children who consume fewer family meals consume more unhealthy food.	Literature Review
Naska et al. (2015)	[42]	23,162 middle-aged European adults	Those who ate more foods outside of the home consumed more sweet and savoury bakery items, soft drinks, juices and other non-alcoholic beverages than those who ate more at home	Cross-Sectional
Neumark-Sztainer et al. (2003)	[13]	4746 adolescents, Project EAT	Frequency of family meals was positively associated with intake of fruits, vegetables, grains, and calcium-rich foods and negatively associated with soft drink intake. Frequency of family meals was associated with consumption of energy, protein, calcium, iron, folate, fiber, and vitamins A, C, E, and B6.	Cross-Sectional
O’Dwyer et al. (2005)	[10]	958 Irish adults aged 18–64	Intakes of fiber, micronutrients, calories, protein, fat and carbohydrates were greater at home than away from home.	Cross-Sectional
Patrick and Nicklas (2005)	[47]	Families (with children)	Children who eat meals with their families generally consume more healthy foods and nutrients. Eating out is associated with higher intake of fat and calories than eating at home.	Literature Review
Poti and Popkin (2011)	[48]	29,217 children aged 2–18 (national sample)	Between 1977 and 2006, children had an overall increase in energy intake corresponding with a decrease in frequency of eating at home (compared to outside of the home).	Longitudinal
Surjadi et al. (2017)	[49]	6503 children were followed from kindergarten–eighth grade	Family meals in kindergarten and increase in family meal frequency over time both predicted healthier dietary intake in eighth grade among White and Black adolescents, but not among Hispanic or Asian adolescents.	Longitudinal
Sweetman et al. (2011)	[41]	434 children aged 2–5	Frequency of family mealtimes was unrelated to vegetable consumption or liking.	Cross-Sectional
Utter et al. (2008)	[50]	3245 adolescents (national sample)	Frequency of family meals was associated with consuming five fruits and vegetables per day, eating breakfast, and bringing lunch from home.	Cross-Sectional
Videon and Manning (2003)	[51]	18,177 adolescents (national sample)	Parental presence at family meals was associated with greater consumption of fruits, vegetables, dairy foods, and breakfast.	Cross-Sectional
Woodruff and Hanning (2008)	[52]	Families (with adolescent children)	Family meals were generally associated with improved dietary intake.	Systematic Review
Woodruff & Hanning (2009)	[53]	3223 Canadian middle school students	Frequency of family meals was associated with breakfast consumption and decreased consumption of soft drinks.	Cross-Sectional

**Table 3:** Summary of Psychosocial Outcomes.

Author(s) and Year	Reference Number	Sample	Main Outcomes	Study Design
Psychosocial Outcomes				
Eisenberg et al. (2004)	[5]	4746 adolescents, Project EAT	Frequency of family meals was related to decreased risk for depressive symptoms, low GPA, suicide involvement, and tobacco, alcohol, and marijuana use after controlling for family connectedness.	Cross-Sectional
Eisenberg et al. (2008)	[7]	806 adolescents, Project EAT	Family meal frequency at age 12 was associated with lower odds of smoking, alcohol use, and marijuana use at age 17 for females. Family meals were not associated with substance use for males.	Longitudinal
Franko et al. (2008)	[28]	2379 females tracked annually from age 9–19	More frequent family meals from ages 9–11 predicted lower likelihood of smoking during adolescence.	Longitudinal
Fulkerson et al. (2009)	[14]	Racially diverse sample of 145 adolescents who attended alternative high school	Family dinner frequency was negatively associated with depressive symptoms.	Cross-Sectional
Goldfarb et al. (2014)	[6]	Adolescents	Family meals were associated with decreased risk for alcohol and marijuana use, sexual activity, depression and suicidal behaviors and ideation, violence and delinquency, and increased overall wellbeing, but these associations were less likely to be statistically significant when measures of family connectedness were included.	Systematic Review
Lu et al. (2011)	[67]	160 non-obese adult women	Meals eaten at home were followed by less worry and more positive emotions than meals eaten away from home.	Observational
Neumark-Sztainer et al. (2004)	[68]	4746 adolescents, Project EAT	Adolescents who reported frequent family meals, high priority for family meals, and more structure in family meal environment were less likely to engage in disordered eating.	Longitudinal
Sen (2010)	[66]	6748 children aged 12–16 (national sample)	Frequency of family meals was related to decreased substance use and running away for females, and decreased incidence of drinking, physical violence, property destruction, stealing, and running away from home for males.	Cross-Sectional
Skeer and Ballard (2013)	[57]	Families with adolescent children	Frequent family meals were associated with decreased substance use, aggressive and violent behavior, poor school performance, sexual behavior, mental health problems, and disordered eating behaviors.	Literature Review

**Table 4:** Summary of Family Relationships.

Author(s) and Year	Reference Number	Sample	Main Outcomes	Study Design
Family Relationships				
Czaja et al. (2011)	[70]	74 families with children aged 8–13	Children who demonstrated loss of control eating were more likely to come from families that had less healthy patterns of communication, interpersonal involvement, and family functioning during an observed family meal than children who did not have loss of control eating.	Observational
Fiese et al. (2012)	[32]	200 families with children aged 5–12	Families with overweight or obese children spent less time on a family meal and spent less time in positive communication than families with children of a healthy weight.	Observational
Fiese (2006)	[69]	Families with children	Family meals are one family routine that promote health and wellbeing, in part through the positive contributions they make to family relationships and functioning.	Review/Synthesis
Franko et al. (2008)	[28]	2379 females tracked annually from age 9–19	More frequent family meals from ages 9–11 predicted greater family cohesion and coping skills when children were 18–19 years old.	Longitudinal
Fulkerson et al. (2006)	[72]	902 adolescents, Project EAT	Adolescents and parents both reported high levels of communication and enjoyment of family meals. Younger adolescents reported higher importance of eating together than older adolescents.	Cross-Sectional
Fulkerson et al. (2010)	[26]	4750 racially diverse, low-income adolescents	Frequency of family meals was associated with adolescent perceptions of parent–child communication over time.	Longitudinal
Welsh et al. (2011)	[54]	152 adults and 75 adolescents from 90 households	Family meal frequency was positively correlated with perceived family cohesion.	Cross-Sectional

## Data Availability

No new data were created or analyzed in this study. Data sharing is not applicable to this article.
